# Supplementary figures and images for: Molecular Assessments, Statistical Effectiveness Parameters and Genetic Structure of Captive Populations of Tursiops truncatus Using 15 STRs
Source: Animals (Basel). 2022 Jul 21;12(14):1857. doi: 10.3390/ani12141857 (PMC9312175; doi:10.3390/ani12141857)

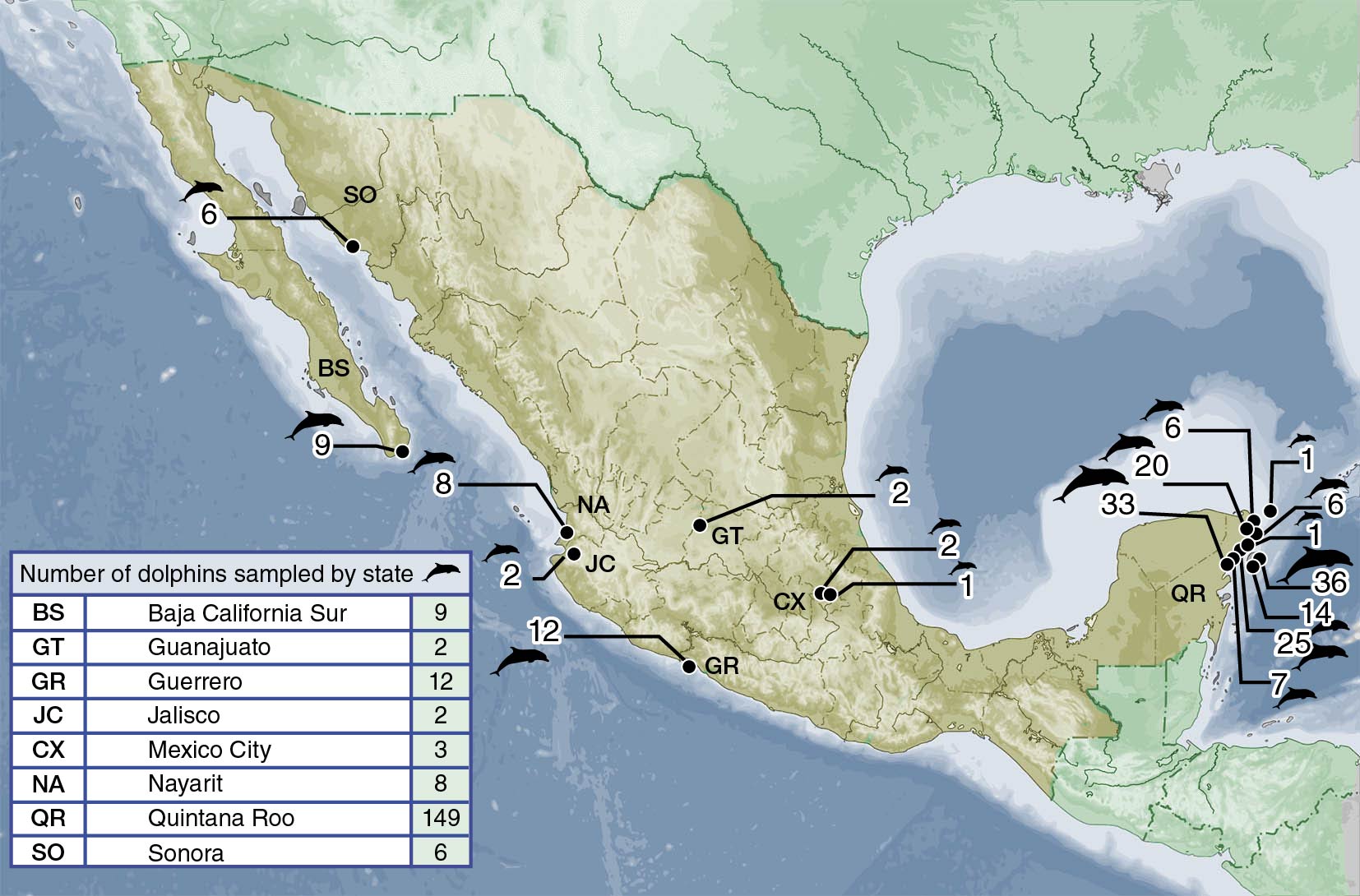

Supplement: Supplementary file 1 [file animals-12-01857-s001.zip › Figure S1.jpg]
